# Scutellarin’s Cardiovascular Endothelium Protective Mechanism: Important Role of PKG-I*α*

**DOI:** 10.1371/journal.pone.0139570

**Published:** 2015-10-06

**Authors:** Lin Li, Lu Li, Chen Chen, Jian Yang, Jiaxun Li, Na Hu, Yang Li, Dongmei Zhang, Tao Guo, Xuan Liu, Weimin Yang

**Affiliations:** 1 School of Pharmaceutical Science & Yunnan Key Laboratory of Pharmacology for Natural Products, Kunming Medical University, Kunming, China; 2 Department of Cardiology, First Affiliated Hospital of Kunming Medical University, Kunming, China; 3 Shanghai Institute of Materia Medica, Chinese Academy of Sciences, Shanghai, China; Emory University, UNITED STATES

## Abstract

Scutellarin (SCU), a flavonoid glycoside compound, has been successfully used in clinic for treatment of ischemic diseases in China. In this report, we checked the effects of SCU on endothelium dysfunction (ED) of coronary artery (CA) against myocardial ischemia reperfusion (MIR) injury *in vivo*. The involvement of PKG-Iα was further studied using cultured endothelial cells subjected to hypoxia reoxygenation (HR) injury *in vitro*. In rat MIR model, SCU (45 and 90 mg/kg, iv) significantly reduced ischemic size and restored the endothelium-dependent vasodilation of isolated CA rings. PKG inhibitor Rp-8-Br-cGMP (50 μg/kg, iv) could ameliorate the protective effects of SCU. Increase in phosphorylation of vasodilator-stimulated phosphoprotein (VASP), a main substrate of PKG, at Ser 239 was observed in both heart tissue and serum of SCU-treated animals. In cultured human cardiac microvascular endothelial cells (HCMECs), SCU (1 and 10 μM) dose-dependently protected cell viability and increased the mRNA and protein level of PKG-Iα against HR injury. The activity of PKG was also increased by SCU treatment. The activation of PKG–1α was then studied using targeted proteomic analysis (MRM-MS) checking the phosphorylation state of the autophosphorylation domain (aa42-94). Significant decrease in phosphorylation of PKG-Iα at Ser50, Ser72, Ser89 was induced by HR injury while SCU treatment significantly increased the phosphorylation of PKG-Iα, not only at Ser50, Ser72 and Ser89, but also at Ser44 and Thr58 (two novel phosphorylation domains). Our results demonstrate PKG-Iα might play an important role in the protective effects of SCU on ED against MIR injury.

## Introduction

Scutellarin (SCU) is a flavonoid glycoside compound (4’, 5, 6-trihydroxy flavonoid-7-glucuronide) isolated from the traditional Chinese medicine plant *Erigeron breviscapus (Vant*.*)* Hand. Mazz [1]. Both purified SCU and plant extract mainly containing SCU had been successfully used in China in clinic. Oral or injectable forms of SCU preparations had been developed for the treatment of ischemic cardiovascular diseases [1–2]. Reported pharmacological activities of SCU include anti-oxidative [3], anti-inflammatory [4], vasodilative [5], anti-apoptosis [6–7] and angiogenesis activities [8]. Especially, the protective effects of SCU against ischemic injuries such as myocardial ischemia reperfusion (MIR) injury [6, 9] have attracted interests of researchers, though the mechanism of SCU was still not fully clarified.

MIR injury would result in endothelium dysfunction (ED) because endothelium is an early active participant in IR injury, and endothelial cells are particularly susceptible to IR injury [10–14]. In IR injury, structural and functional abnormality of endothelial cell occurs earlier than that of parenchyma cell, while the functional recovery of endothelial cell occurs at a later stage [15]. Vascular ED is also a major contributor to the development and exacerbation of many cardiovascular diseases [10]. In particular, ED can lead to endothelium dependent vasodilation impairment, proliferation of vascular smooth muscle cell and excessive vasoconstriction [12]. Besides endothelial cell, vascular smooth muscle cell dysfunction also contributes to ED [15]. ED is characterized by impaired endothelium-dependent vasorelaxant response to acetylcholine (ACH) in which cGMP dependent protein kinase (PKG) is involved [16]. Damaged endothelium reduces perfusion to areas of previous ischemia and thus exacerbate organ damage [17]. Therefore, endothelium plays a critical role in determining the organ damage extent resulted from IR and interventions capable of protecting the endothelium from IR would be of great clinical interest.

The eNOS/cGMP/PKG pathway plays a key role in endothelium-dependent relaxation. Endothelial NO generated from L-arginine via the activation of endothelial NO synthase (eNOS) activates guanylate cyclase and produces cGMP, which leads to PKG-mediated vasodilation [18–20]. In our previous study, SCU was found to induce endothelium-dependent vasodilative effect partially affected by inhibitors of NOS and guanylate cyclase in isolated mouse aorta [21]. In the present study, we focused on investigating whether SCU have direct cytoprotective effects on endothelial cells against IR injury. The effects of SCU on ED of coronary artery (CA) in a rat MIR injury model were checked and the role of PKG in the effects of SCU was studied. *In vitro* model of cultured human cardiac microvascular endothelial cells (HCMECs) subjected to simulated hypoxia reoxygenation (HR) injury was used. And, phosphorylation of vasodilator-stimulated phosphoprotein (VASP) at Ser 239 was used to monitor PKG activity. After finding PKG–1α as a possible target of SCU, the phosphorylation state of PKG–1α was further studied because phosphorylation played an important role in PKG-I activation [22–25]. Recent development in multiple-reaction monitoring mass spectrometry (MRM-MS) provides a useful tool for thoroughly measuring the absolute quantity of modification such as phosphorylation within interested proteins [26]. To be noted, PKG was reported to be involved in IR injury though its function had not been fully clarified [27–30]. Therefore, the phosphorylation states of PKG-Iα under different treatments were analyzed in the present study using MRM-MS, a targeted proteomic analysis method, to clarify the PKG-Iα function in IR injury and the protective mechanism of SCU.

## Materials and Methods

### Reagents

Powdered SCU (purity 99%, formula weight 464.4) was obtained from Mr. Renwei Zhang of Kunming Longjin Pharmaceuticals Co. (Kunming, China). SCU stock solutions (maximum concentration 100 mM) were prepared by dissolving the SCU in physiological saline solution. Cell culture reagents including modified RPMI–1640 medium and fetal bovine serum were obtained from HyClone (Thermo Fisher Scientific, Waltham, MA, USA). PKG inhibitor Rp-8-Br-cGMPS was purchased from Santa Cruz Biotechnology (Dallas, TX, USA). PKG activator 8-pCPT-cGMP [8-(4-Chlorophenylthio) guanosine 3',5'-cyclic monophosphate sodium salt], triphenyl tetrazolium chloride (TTC) and ACH were purchased from Sigma-Aldrich (St. Louis, MO, USA).

### Study design

In order to study cardiovascular endothelium protective mechanism of SCU and the role of PKG, the investigations were carried out in both MIR rats *in vivo* and in HR cells of HCMECs *in vitro*. In the animal studies, the protective effect of SCU on MIR injury was tested by evaluating myocardial ischemia area, and then the hypothesis that SCU improves endothelium dependent vasodilation reduced by MIR was proved in the animal model with ED induced by MIR, while PKG inhibitor was used to reveal the influence of PKG. In *vivo* animal studies, there were four independent experimental design series. 1) To evaluate the effects of SCU on myocardial ischemia area induced by MIR injury, SD rats were divided into four groups: sham, MIR model and two SCU groups (45 or 90 mg/kg, iv). 2) In another experiment, the influence of PKG inhibitor and SCU on myocardial ischemia area was assessed. The rats were divided into four groups: MIR model, SCU (45 mg/kg, iv), PKG inhibitor (50 μg/kg Rp-8-Br-cGMPS, iv), and SCU (45 mg/kg, iv) + PKG inhibitor (50 μg/kg, iv) treated group. 3) In another independent experiment of MIR, endothelial-dependent vasodilation in CA rings was assayed. Rats were divided into five groups: sham, MIR model, SCU groups (45 mg/kg, iv), PKG inhibitor (50 μg/kg Rp-8-Br-cGMPS, iv) and SCU (45 mg/kg, iv) + PKG inhibitor (50 μg/kg, iv) treated groups. 4) To evaluate the effects of SCU on PKG-I and p-VASP Ser 239 changes induced by MIR injury, SD rats were divided into five groups: sham, MIR model and two SCU groups (45 or 90 mg/kg, iv), nitroglycerin (NTG, 1.5 mg/kg, iv) group (used as positive control).

In *vitro* cell studies, HR treatment was used to simulate *vivo* IR injury. To investigate whether SCU has direct cytoprotective effects and the role of PKG, cell viability and protein, activity and mRNA of PKG were determined. In addition, the phosphorylation site of PKG–1α was further studied using MRM-MS to clarify how SCU affects the phosphorylation of PKG activation. In *vitro* studies, cells of HCMECs were incubated with SCU (0.1–10 μM) under normal culture and simulated HR conditions. In each assay, three independent experiments (each conducted in triplicate) were used.

### MIR model and evaluation of ischemia area

Adult Sprague-Dawley (SD) rats (180–220 g) were provided by the animal center of Kunming Medical University. The rats were housed under controlled temperature (23–25°C) and lighting (8:00–20:00 light, 20:00–8:00 dark) with free access to food and drinking water. The study and the protocol were approved by the Animal Care and Use Committee of Kunming Medical University and conformed to the standards set by the Yunnan Experimental Animal Management Board. The animal procedures were performed conform the NIH guidelines (Guide for the care and use of laboratory animals). For anesthesia, 10% urethane (1mL/100 g with dose of 1g/kg) in saline solution was intraperitoneally injected to the rats. Before initiating surgery, to render the animal areflexic to strong ear- or tail-pinch, if needed, additional doses of ≤0.4 ml 10% urethane were used. SCU and other treated drug infusion was initiated intravenously 15 min prior to MIR surgery via the tail vein (2 mL/h) until the ending of reperfusion. The sham and MIR groups were given normal saline (NS).

The MIR model was induced according to the previously described procedure with minor modifications [31]. The anesthetized rats were intubated and ventilated using a small-animal ventilator. Each rat was ventilated with room air at a tidal volume of 2 mL per 300 g and a rate of 70 breaths/min. Left thoracotomy was performed at the fourth intercostal space and the heart was exposed. The left anterior descending (LAD) CA was encircled 3–4 mm from its origin with a 5–0 silk suture for subsequent LAD occlusion. The LAD was then ligated for 40 min to induce myocardial ischemia, followed by removal of the ligation to allow subsequent reperfusion for 120 min. Sham-operated rats were subjected to the same surgical procedures, except the suture under the LAD was not tied. Euthanasia was performed by intraperitoneal injection of 100 mg/kg thiopental at the end of the experiment, the hearts were then excised and the atria and right ventricular free wall dissected off the left ventricle.

The hearts were frozen and stored in a −20°C freezer for 5 min, and then sliced into 1-mm-thick transverse sections across the long axis. Slices were incubated in 1% TTC in a phosphate buffer (pH 7.4) for 15 min at 37°C. After staining, the slices were immersed in 4% formalin. The tissues that stained brick red were considered viable, whereas pale or white tissues were considered necrotic. The infarct areas were analyzed by Image-Pro Plus 6.0 (IPP) software, and the ischemic area was expressed as a percentage of the area at risk: infarct size = 100×infarct area (white area)/risk area (white area + red area). Infarct size was expressed as the percentage of the total cross-sectional area of the ventricles. In addition, to measure PKG and p-VASP in heart tissue and serum, the infarct section of heart and serum sample were collected and wrapped in foil and stored in liquid nitrogen for Western blotting analysis.

### Evaluation of ED in isolated CA rings from MIR rats

The treated rats were sacrificed and the left heart placed in a dissection dish filled with ice-cold (4°C) PSS solution (in mmol/L): NaCl, 140.0; KCl, 4.7; CaCl_2_, 1.6; MgSO_4_, 1.2; 3-[N-morpholino]-propanesulfonic acid (MOPS), 1.2; Na_2_HPO_4_, 1.4; EDTA, 0.02; D-glucose, 5.6; pH adjusted to 7.4 at 37°C. KCl (60 mmol/L)-PSS is a substituted PSS in which NaCl has been substituted by KCl (60 mmol/L) in an equal molar fashion.

An integrated wire myograph system (Model 620M, DMT-Asia Ltd, Shanghai, China) was applied with a Motic SMZ168-TL stereomicroscope to dissect the blood vessels. The CA of LAD was dissected free, adhering connective tissue cleaned off, and cut the segments following the point of occlusion into 1-mm-long ring. The rings were mounted with 60-μm steel wires in separated tissue baths of the wire myograph system for tension recording. The tissue baths were filled with PSS solution (pH 7.4) at 37±1°C and aerated with O_2_; washout was performed by draining and replacing the bathing solution using a syringe.

Isometric tension signals were recorded and data collected by a Powerlab data acquisition system (AD Instruments Asia, Shanghai, China). Each ring was stretched to an optimal tension of 1 mN and permitted to equilibrate for 90 minutes before the experiment started. Thirty minutes after setting up the wire myograph, each ring was initially contracted by KCl (60 mmol/L)-PSS and washed and the viability of the rings measured. Rings with twice amplitude of contraction less than 10% were used for the experiment. The rings were contracted by U46619 (1 μmol/L) and relaxed using cumulative addition of ACH (0.001–100 μmol/L) to test the endothelial-dependent vasodilation by calculating EC_50_ and E_max_ values.

### Cell culture and simulated HR injury

HCMECs were obtained from the Shanghai Yansheng Biochemical Technology Company (Shanghai, China) and grown in 1640 medium supplemented with 10% fetal bovine serum and 1% penicillin/streptomycin antibiotics. Tightly confluent monolayers of HCMECs from 4th-15th passage were used in all experiments. In experiments checking the effects of SCU under normal condition, cells were treated with vehicle control (NS) or SCU at different concentrations or PKG activator (8-pCPT-cGMP, 30μM) for 26 h. In experiments checking the effects of SCU under HR condition, cells were incubated with SCU at different concentrations or PKG activator (8-pCPT-cGMP, 30μM) for 2 h prior to HR injury and during HR injury (24 h). Control cells were cultured in parallel and kept in normal culture condition for the entire time period (26 h). Simulated HR injury was induced according to previously described procedures [29] with minor modifications. Briefly, HCMECs were placed in a humidified hypoxic chamber (HF100, Heal Force Biotech Co, Shanghai, China) for 12 h of hypoxia (5% CO_2_+ 2% O_2_ + 93% N_2_) with medium free of glucose and serum at 37°C, followed by 12 h of re-oxygenation (5% CO_2_+ 95% air) in complete medium containing glucose and serum. At the end of the experiment, cell viability was examined using MTT assay as described below. Furthermore, cells of each group were also collected for real-time PCR assay, western blotting and ELISA assay as described in following sections.

### MTT and Trypan blue staining assay of cell viability

For MTT assay, cells were plated in 96-well flat-bottomed plates at a density of 3×10^4^ cells/ml and 90μl/well. Cells were cultured in normal culture condition or treated with simulated HR injury as described above. After the treatments, 20 μl of MTT (5 mg/ml) was added to each well and the plates were incubated for 4 h at 37°C. Then, 100 μl of lysis buffer (20% sodium dodecyl sulfate [SDS] in 50% N,N-dimethylformamide, containing 0.4% [v:v] 1N HCL and 0.5% [v:v] 80% acetic acid) was added to each well and incubated overnight. Cell viability was determined by measuring the ability of metabolically active cells to convert the yellow tetrazolium salt MTT into purple formazan crystals with a Microplate Reader at 570 nm. Results of three independent experiments (each conducted in triplicate) were used for statistical analysis. Cell counting and trypan blue exclusion viability was performed on Countstar IC 1000 (Inno-Alliance Biotech) automated cell counter and the percentage of viable cells was obtained.

### RNA extraction and real-time PCR analysis

Total RNAs were extracted using the TRIzol® reagent (Invitrogen) and reverse transcribed using PrimeScript® RT reagent Kit with gDNA Eraser (TaKaRa) according to the manufacturer’s instructions. Real-time PCR amplifications was performed using SYBR® Premix Ex TaqTM II (TaKaRa) and the thermal profile was 95°C for 2 min followed by 40 cycles at 95°C for 15 sec and 61°C for 20 sec. Each sample was measured in triplicate and the mean threshold cycle (Ct) value was calculated. Relative expression was calculated using the ΔΔCt method. The mRNA expression of GAPDH was used as an internal control. Primer sequences used for PKG-Iα analysis were 5’- GGCTGTCAGAGAAGGAGGAAG–3’ and 5’-GGAAGGACCTGTACGTCTGC–3’. Primer sequences used for GAPDH analysis were 5’-GGAGCGAGATCCCTCCAAAAT–3’ and 5’-GGCTGTTGTCATACTTCTCATGG–3’.

### Western blotting assay

For rat heart tissue homogenate samples, the preparation comprises following steps: after removal from liquid nitrogen, tissue wrapped in foil were hammered and transferred into a precooled glass homogenizer containing 200 μL precooled RIPA buffer and homogenized for 10 min, transferred to Eppendorf tube, and centrifuged at 18,000 rpm for 10 min at 4°C. The supernatants were collected and stored at −80°C until Western blotting analysis. For cell samples, cells were lyzed in RIPA buffer containing 50 mM Tris HCl (pH 7.4) with 150 mM sodium chloride, 1.0% Nonidet P–40, 1% sodium deoxycholate, 1.0% Triton X–100, 1 mM EDTA, and protease inhibitor cocktail (Roche Diagnostics. Indianapolis,IN,USA). After centrifugation, the supernatant were collected and stored at −80°C until Western blotting analysis.

Protein concentrations were determined using a BCA Protein Assay Kit (Beyotime Biotechnology, Haimen, Jiangsu, China). The protein samples were denatured by mixing with equal volume of 2 × sample loading buffer and then boiling at 100°C for 5 min. An aliquot (20 μg as protein) of the supernatant was loaded onto a 10% SDS gel, separated electrophoretically, and transferred to a polyvinylidene fluoride membranes (Millipore, Billerica, MA, USA). After the membrane was incubated with 10 mM TBS with 20% Tween 20 and 5% dehydrated skim milk to block nonspecific protein binding, the membrane was incubated with primary antibodies overnight at 4°C. The primary antibodies used were rabbit anti-PKG-I polyclonal antibody (C8A4, 1:1000, Cell Signaling Technology, Danvers, MA, USA), rabbit anti-vasodilator-stimulated phosphoprotein (VASP) (1:500. Abcam, Cambridge, MA,USA), rabbit anti-phosphorylated-VASP (p-VASP) (Ser239, 1:500. Santa Cruz Biotechnology, Dallas, TX, USA) and rabbit anti-GAPDH. After TBS washes, blots were then incubated with secondary antibody for 1 h at room temperature at a 1:5000 dilution and then visualized using enhanced chemiluminescence (ECL) kit (GE Healthcare, Buckinghamshire, UK). The secondary antibodies used were horseradish peroxidase (HRP)-coupled anti-rabbit secondary antibody (Santa Cruz Biotechnology). The density values of bands were quantified by densitometric analysis of scanned images (Scion image 4.03). The relative protein ratio was calculated by determining the integrated intensity of the bands of each treated group as a ratio of the control condition.

### ELISA assay of PKG activity in HCMECs

An enzyme-linked immunosorbent assay (ELISA) kit for PKG activity was supplied by CycLex Co., Ltd **(**Nagano, Japan). The kit is primarily designed to measure the activities of PKG (including PKG-I and–II), and in which the phosphor-specific monoclonal antibody used has been demonstrated to recognize the phosphor-threonine 68/119 residues on substrate of PKG. Collecting HCMECs under normal and HR condition, determined the activities of PKG in the cells following the kit procedures.

### Targeted proteomic analysis of phosphorylation state of PKG-I α

Cells were divided into four groups including control, HR model, SCU 1 μM, HR+SCU 1 μM group and underwent different treatments as described above. After treatments, cells were washed with ice-cold PBS and then scraped off with a cell scraper. After centrifugation for 5 min at 1500 g, the supernatant was discarded and the cell pellet was resuspended in lysis buffer containing 7 M urea, 2 M thiourea, 2% CHAPS and 1% DTT, 0.8% Pharmalyte, a mixture of Protease inhibitors (all from Bio-Rad) and incubated 30 min on ice. The lysed cells were centrifuged at 15000 g for 30 min at 4°C and the supernatants were collected for analysis. Protein sample (1mg) was reduced by incubating with 50 μl 100 mM DTT in 100mM ammonium bicarbonate for 45 min at 56°C. After cooling to room temperature, alkylation was induced by adding 250 μl 200mM IAA and incubating in darkness for 30 min at room temperature. After alkylation, equal volume of acetone was added to precipitate protein at -20°C for 30 min. After centrifugation at 15000 g at 4°C for 15 min, the supernatant was discarded and the protein pellet was washed twice by 75% alcohol. The protein sample was dried down by evaporation at room temperature in a vacuum concentrator/centrifugal evaporator and then dissolved in 1 ml 50 mM ammonium bicarbonate buffer. Digestion was conducted by adding 20 μg trypsin (1:50 enzyme:protein ratio) at 37°C overnight. After drying by evaporation at room temperature in a vacuum concentrator/centrifugal evaporator, the sample was redissolved with 900 μl 0.1% formic acid and the desalted by the Waters sep-pak Vac C18 Spin Column (Waters Corporation milford, Massachusetts USA). The sample was dried by evaporation at room temperature in a vacuum concentrator/centrifugal evaporator and then stored at -80°C until mass spectrography (MS) analysis.

The nanoHPLC-ESI MS/MS analysis was performed with a 4000 Q TRAP system (AB Sciex, USA) operated in the positive mode using the Nanospray® III Source and heated interface. Briefly, each protein sample (5 μl) was eluted onto a C18 reversed phase column (PepMap100 C18, 75 μm x 5μm x 15cm) at a flow rate of 300 nl/min. Peptides were separated using a 60 min gradient ranging from 5% to 50% mobile phase B (mobile phase A: 2% acetonitrile, 0.1% formic acid; mobile phase B: 98% acetonitrile, 0.1% formic acid). By using the MRM Pilot software (AB Sciex, USA) to predict the mass signals which might be produced by the hydrolysates, a MRM initiated detection and sequencing scan (MIDAS) method was developed for detection of PKG-I α phosphopeptides in sequence of aa42-94. The MS analysis was performed on the 4000 Q TRAP system in information dependent acquisition (IDA) mode and the setting parameters were: ion spray voltage: 2300 V; curtain gas: 20; ion source gas: 15; and heated interface: 150°C. Protein samples from three independent experiments were analyzed. And, for each experiment of protein samples, triplicate technological repeat were performed to ensure reproducibility. The detected amounts of PKG I α phosphopeptides (peak areas) were further normalized using internal standard. The two un-phosphorylated peptides of PKG I alpha used as internal standards were ILMLKEER (516.3/2+) and EEEIQELKR (587.3/2+), aa10-17 and aa29-37, respectively. Briefly, the raw peaks were processed, and extracted ion chromatograms (XIC) were generated from the full-scan MS using MultiQuant 2.0.2 (AB Sciex, USA). Peak areas of PKG Iα phosphopeptides were normalized by peak areas of tryptic digested peptides of internal standards. The normalized phosphorylation rates were calculated as ratio of peak area of PKG I alpha phosphopeptides to peak area of the average of two un-phosphorylated peptides of PKG I alpha.

### Statistical analysis

Values were expressed as mean ± SEM. Statistical analysis was performed using statistical software Sigma Stat 3.1. Nonlinear regression analysis for individual concentration-response curves was performed using a Hill algorithm in Sigma Plot 10.0, allowing for an individual geometric ‘‘EC_50_” value to be calculated. The E_max_ value represents the maximal vasodilative response (the minimal relative stress of pre-contraction). Comparisons were made using one-way ANOVA, two-way ANOVA analysis or unpaired t-test. *P*< 0.05 was considered statistically significant.

## Results

### Protective effects of SCU in rat MIR injury model and influence of PKG inhibitor on the effects of SCU

**Fig 1:**
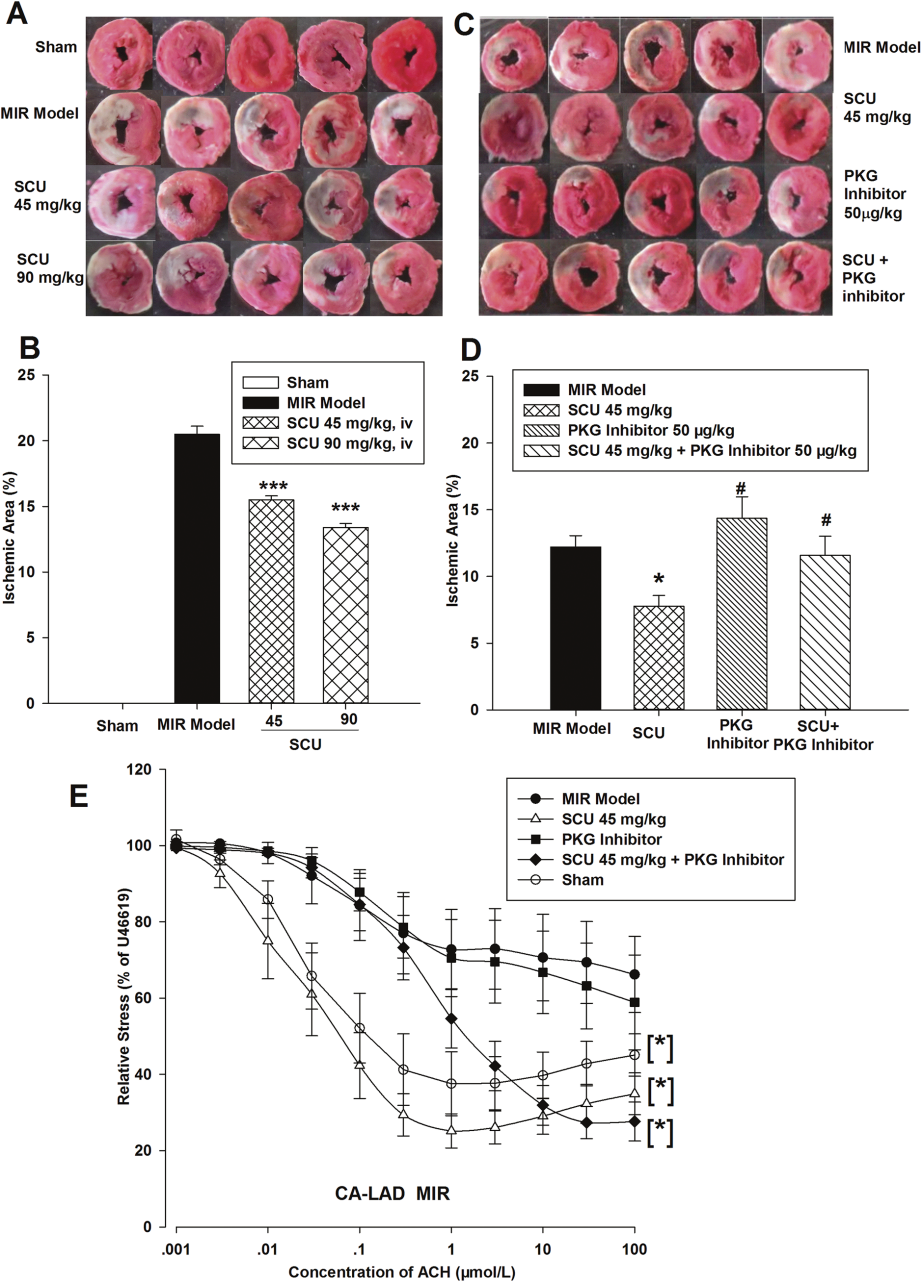
SCU decreases ischemia area, improves endothelium-dependent vasodilation and influence of PKG inhibitor in MIR rats. (A) Representative images of TTC staining of ischemic heart slices from myocardial MIR rats (ischemia 40/reperfusion 120 minutes). (B) Quantification of the myocardial ischemia area in rat hearts. n = 10 rats in each group. One-way ANOVA followed by SNK test vs. Model, ***P<0.001. (C) Representative images of TTC staining of ischemic heart slices from myocardial MIR rats (ischemia 40/reperfusion 120 minutes). (D) Quantification of the myocardial ischemic area in rats administered SCU (45 mg/kg, iv) and PKG inhibitor Rp-8-Br-cGMPS (50 μg/kg, iv) alone and co-administered SCU and PKG inhibitor. n = 10 rats in each group. One-way ANOVA followed by SNK test. *P<0.05 *vs*. Model; ^#^ *P*<0.05 *vs*. SCU. The data of the experiments shown in A and B belong to the data of the experiments shown in C and D. (E) Cumulative concentration response curves for acetylcholine chloride (ACH) in isolated CA left anterior descending segments from MIR, SCU-treated (45 mg/kg, iv), and PKG inhibitor Rp-8-Br-cGMPS-treated (50 μg/kg, iv) rats. ACH-induced relaxation is expressed as a percent of pre-contraction by U46199 (1 μmol/L). n = 8–12 segments obtained from 3–4 rats with different treatments. Two-way ANOVA test vs. Model, *P<0.001. Data are presented as mean±SEM.

To evaluate the effects of SCU on MIR injury and the role of PKG, myocardial ischemia area and endothelial-dependent vasodilation (as an ED index) were assessed. As shown in Fig 1A and 1B, SCU treatment (45 and 90 mg/kg, iv) could dose-dependently exhibit cardioprotective effects against MIR injury in rats. The ischemia areas in SCU treated groups were significantly lower than that of the MIR model group. To check whether PKG pathway played role in SCU (45mg/kg, iv) protective effects, the influence of co-treatment of PKG inhibitor with SCU was observed. As shown in Fig 1C and 1D, PKG inhibitor Rp-8-Br-cGMP (50 μg/kg, iv) alone had no significant influence on the ischemic area, but co-treatment of PKG inhibitor with SCU inhibited the cardioprotective effects of SCU.

Results of assay of endothelial-dependent vasodilation in CA (Fig 1E and Table 1) indicated that endothelial function was inhibited in MIR model group. CA from MIR group could not exhibit normal response of relaxation under ACH induction. The fact that CA from SCU treated groups exhibited a response to ACH induction similar to that of sham group indicated that SCU treatment protected endothelium function from MIR injury. Treatment of PKG inhibitor alone did not improve the response of CA to ACH compared to the MIR group (Fig 1E and Table 1). While PKG inhibitor was co-administered with SCU, it significantly right-shifted the ACH dilation curves compared to SCU alone, blocked SCU-induced improvement in ACH-dilation, and increased the EC_50_ of ACH (Table 1).

**Table 1:** Effect of SCU on endothelial vasodilation and influence of PKG inhibitor in CA rings from MIR Rats.

Groups	Dosagemg/kg, iv	n	EC_50_(μmol/L)	E_max_(% of U46619)
Sham	NS	12	0.16±0.12	37.54±8.38*
Model	NS	11	NA	66.19±9.98
SCU	45	8	0.05±0.17	25.16±4.47*
PKG Inhibitor	50 μg/kg	10	NA	58.88±12.42^#^
SCU +PKG Inhibitor	45 mg/kg +50 μg/kg	12	1.29±0.46^#^	27.38±4.24*

Data are means±SEM. PKG inhibitor, Rp-8-Br-cGMPS; E_max_, the maximal vasodilative effect; n, number of CA segments, in each group blood vessel rings were obtained from 3–4 rats with different treatments; NA, not available. NS, normal saline. ^#^ *P*<0.05, one-way ANOVA on Rank Kruskal-Wallis test, EC_50_ *vs*.SCU

*P<0.05 one-way ANOVA followed by SNK test, E_max_ *vs*. Model

^#^ *P*<0.05 E_max_ *vs*. SCU.

### Effects of SCU on p-VASP Ser239 and PKG-I protein level in MIR rats

**Fig 2:**
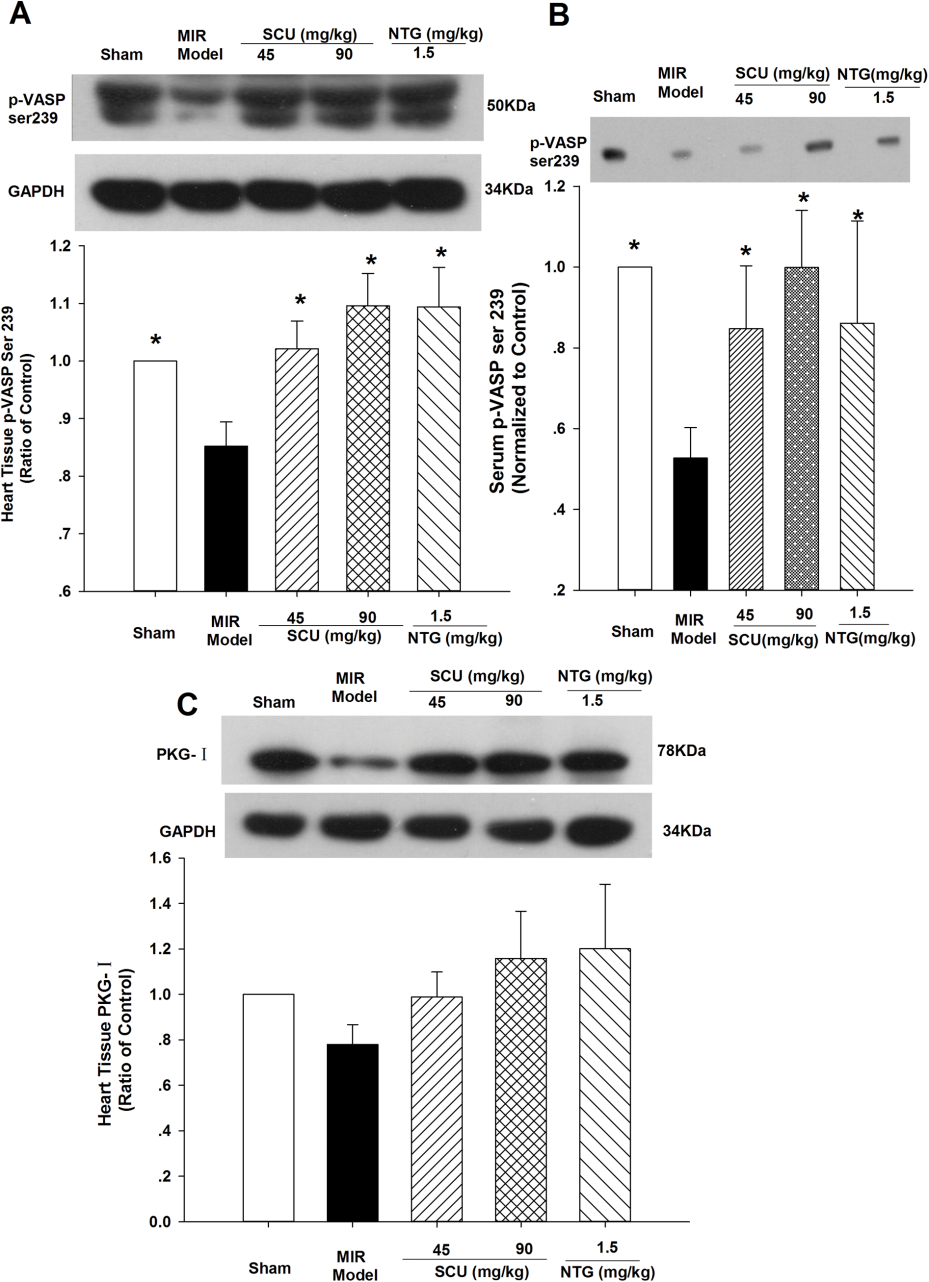
Effect of SCU on PKG-I and p-VASP Ser239 in MIR rats. Representative immunoblot and quantification of p-VASP Ser239 protein level in heart tissue homogenate **(A)** and serum **(B)** from MIR (ischemia 40/reperfusion 120 minutes) rats. (**C)** Representative immunoblot and quantification of PKG-I protein in heart tissue homogenate from MIR rats. n = 10 rats in each group, nitroglycerin (NTG) was applied as positive control. The protein ratio was calculated by determining the band-integrated intensity that was normalized by that of GAPDH as a ratio of sham control except that serum p-VASP was only normalized to sham control as GAPDH could not be detected in serum. Data are means±SEM. One-way ANOVA followed by SNK test vs. Model, *P<0.05 compared to model group.

In order to observe the effects of SCU on PKG expression and activity, PKG-I and it’s phosphorylated product p-VASP Ser239 were determined by western blot method. As shown in Fig 2A and 2B, MIR significantly reduced p-VASP Ser239 level in rat heart tissue and serum compared with sham rats. SCU treatment (45 and 90 mg/kg, iv) and positive control NTG (1.5mg/kg, iv) could increase p-VASP Ser239 level in rat heart tissue and serum compared with MIR model. As p-VASP Ser239 is an indicator of PKG activation, the results suggested that activation of PKG was involved in the protective effects of SCU against MIR injury. As shown in Fig 2C, MIR slightly reduced PKG-I protein level in rat heart tissue but without significance when compared with sham rats. SCU treatment (45 and 90 mg/kg, iv) and positive control NTG (1.5mg/kg, iv) increased PKG-I level in rat heart tissue without significance compared with MIR model.

### Protective effects of SCU on cell viability in HCMECs against simulated HR injury

**Fig 3:**
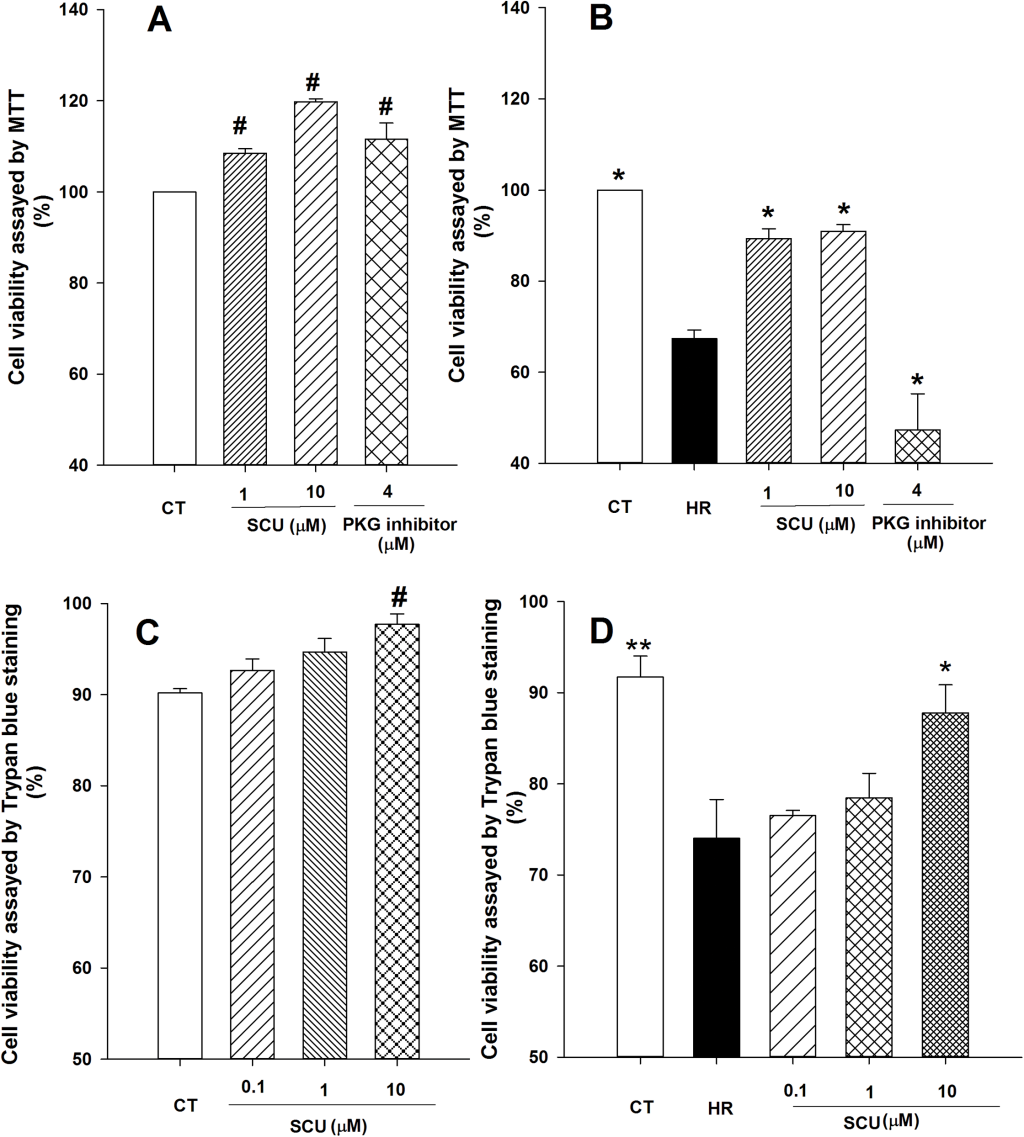
SCU increases cell viability in normal and HR-treated HCMECs. Cell viability was assayed by MTT (**A** and **B**) method and Trypan blue staining (**C** and **D**) under normal (**A** and **C**) and HR treatment (**B** and **D**) culture condition. Experiment under normal culture condition: cells were incubated with SCU for 26 h except control group. One-way ANOVA on Rank followed by SNK test, ^#^ *P*<0.05, compared to control (CT) group. Experiment under HR condition: cells were received HR injury (hypoxia 12/reoxygenation 12 hours) except control group cells and incubated with SCU for 2 h prior to HR injury and following 24 h HR injury. One-way ANOVA followed by SNK test, *P<0.05, **P<0.01 compared to HR group. Data are means±SEM, n = 3 independent experiments with independent culture.

In our preliminary experiments we checked the influence of SCU on cell viability of HCMECs under normal condition, SCU dose <100 μM did not cause significant changes in cell viability while SCU dose >100 μM exhibited cytotoxicity (data not shown). Therefore, SCU at doses of 1 and 10 μM was used in the present study. As shown in Fig 3A, results of MTT assay indicated that SCU (1 and 10 μM) raised cell viability under normal culture condition. Simulated HR injury caused decrease in cell viability of HCMECs, and SCU could partly protect cells from HR-induced injury (Fig 3B). Trypan blue staining proved that SCU (10 μM) significantly increased cell viability under normal (Fig 3C) and HR culture condition (Fig 3D).

### Effects of SCU on mRNA expression of PKG-Iα in HCMECs

**Fig 4:**
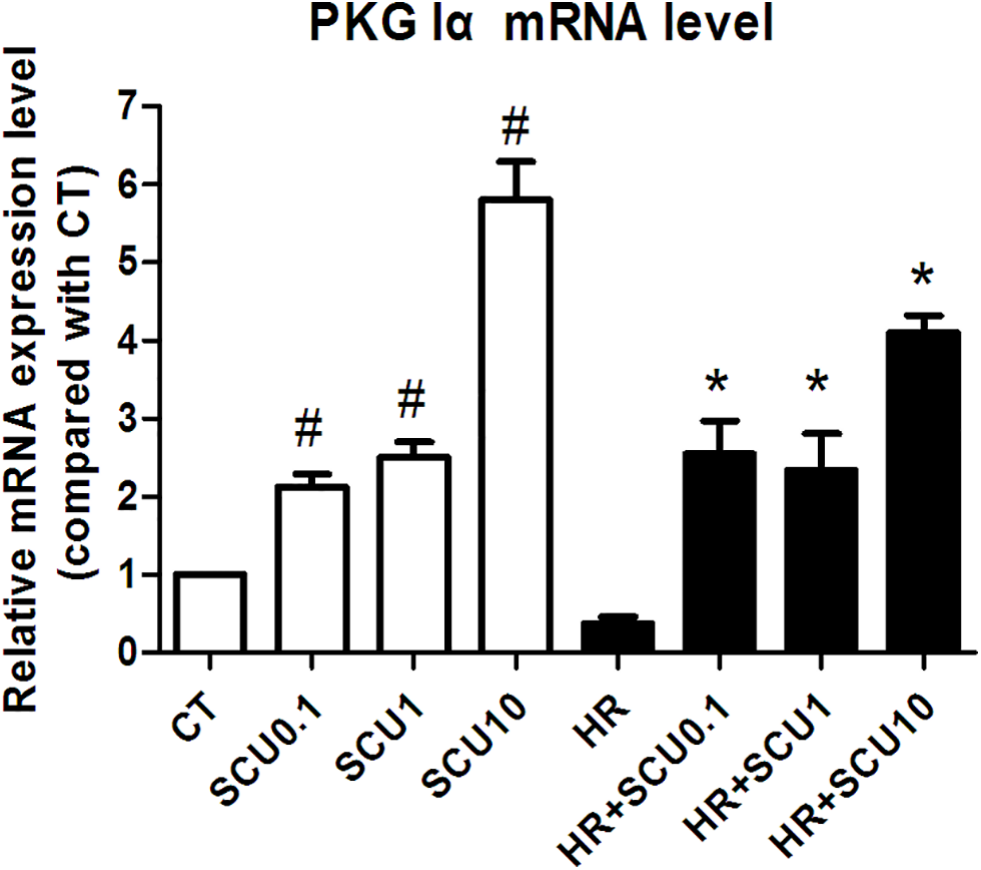
SCU increases PKG-Iα mRNA expression level in normal and HR-treated HCMECs. Quantitative RT-PCR analysis results of PKG I α mRNA expression level in control cells and cells treated with SCU (0.1, 1 and 10 μM) under normal condition or HR injury. Under normal condition, cells were incubated with SCU for 26 h or PBS (CT group). Under IR injury, cells were incubated with SCU or PBS (HR group) for 2 h prior to HR injury (12 h hypoxia followed by 12 h reoxygenation). Data are mean ± SEM, n = 3 independent experiments with independent culture. One-way ANOVA followed by the Fisher LSD test for comparisons was used, ^#^ *p*<0.05 *vs*. control, *p<0.05 *vs*. HR group.

As shown in Fig 4, under normal condition, SCU (0.1, 1 and 10 μM) incubation increased the mRNA level of PKG-Iα. HR injury induced significant decrease in level of PKG-Iα while SCU (0.1, 1 and 10 μM) treatment increased it against HR injury. The results indicated that SCU could up-regulate expression of PKG-Iα and counteract the HR-induced decrease in PKG-Iα mRNA.

### Effects of SCU on PKG protein expression and activity in HCMECs

**Fig 5:**
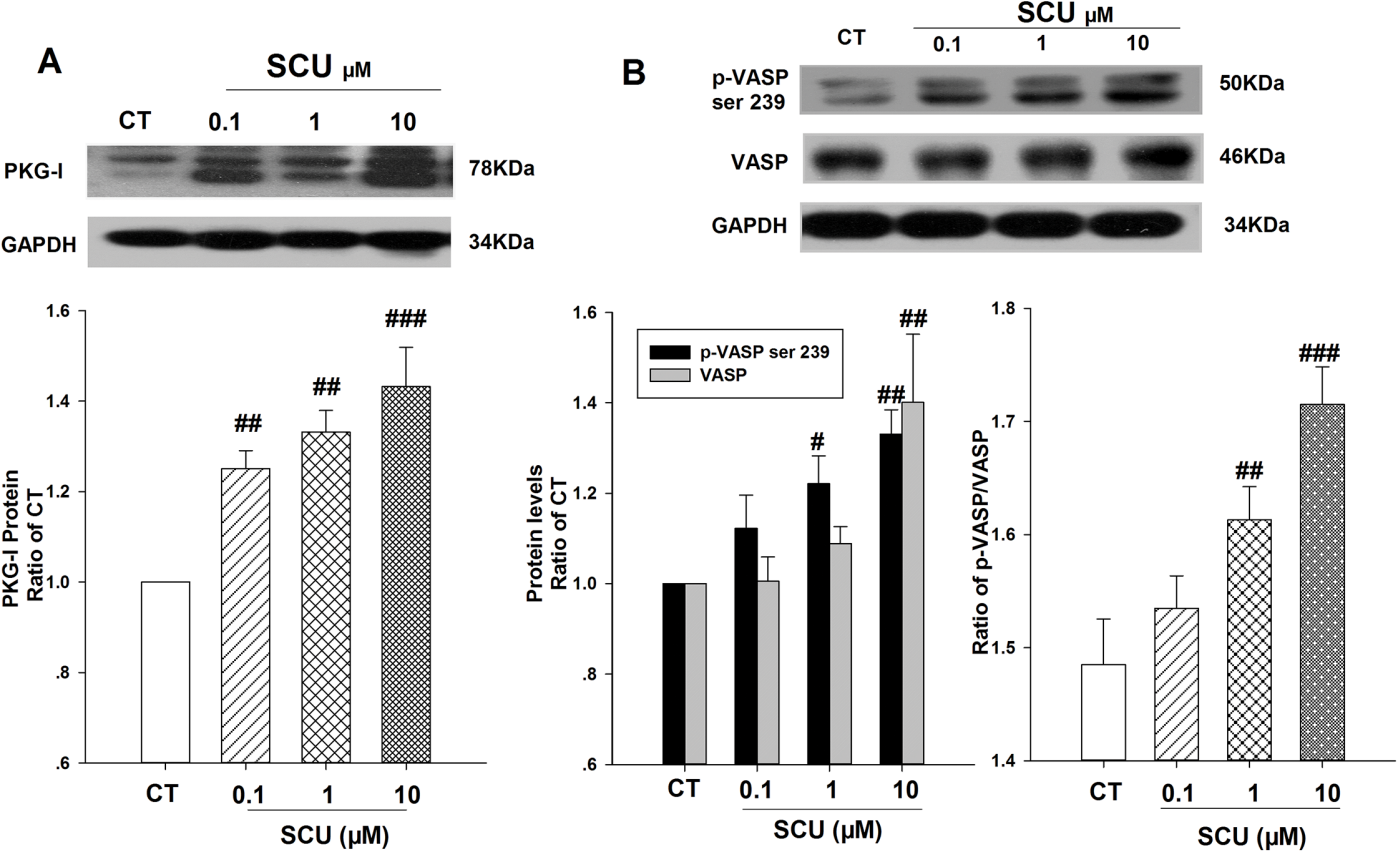
Effect of SCU on PKG-I, VASP, p-VASP Ser239 and ratio of p-VASP/VASP (PKG activity) in normal cultured HCMECs. (A) Representative immunoblot and quantification of PKG-I protein expression in HCMECs. (B) Representative immunoblot and quantification of p-VASP Ser239 and total VASP protein expression in HCMECs. Cells except control group were normally cultured with SCU (0.1, 1 and 10 μM) for 26 h. The protein ratio was calculated by determining the band-integrated intensity that was normalized by that of GAPDH as a ratio of control. Data are means±SEM, n = 3 independent experiments with independent culture. One-way ANOVA followed by Fisher LSD test was used. ^#^ *P*<0.05, ^##^ *P*<0.01 and ^###^ *P*<0.001 compared to control (CT) group.

Since there is no specific antibody for PKG-Iα or PKG-Iβ, Western blotting assay was conducted using antibody against PKG-I. As shown in Fig 5, in normal cultured HCMECs, antibody-against PKG-I identified two protein bands in Western blotting assay. The band with higher molecular weight might be PKG-Iβ (MW 78 kDa) while the band with lower molecular weight might be PKG-Iα (MW 76 kDa). SCU (0.1, 1 and 10 μM) treatment increased the protein level of PKG-Iα (Fig 5A). Furthermore, SCU treatment increased the phosphorylation of VASP at Ser239, which indicated the activity of PKG. Both the levels of p-VASP Ser239 and ratio of p-VASP Ser239 to total VASP in normal cultured HCMECs were increased under SCU treatment (Fig 5B).

**Fig 6:**
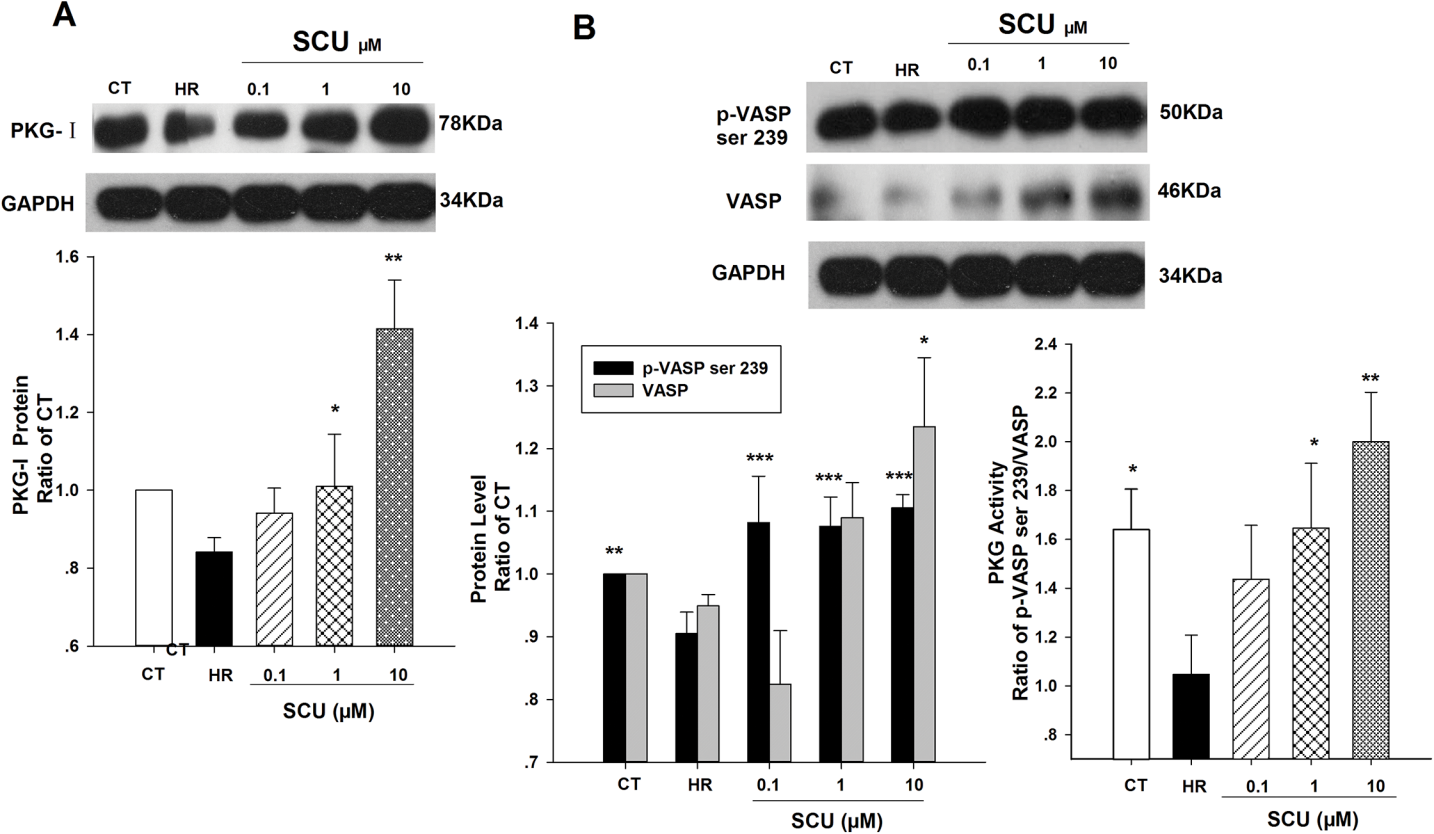
Effect of SCU on PKG-I, VASP, p-VASP Ser239 and ratio of p-VASP/VASP (PKG activity) in HR-treated HCMECs. (A) Representative immunoblot and quantification of PKG-I protein expression in HCMECs. (B) Representative immunoblot and quantification of p-VASP Ser239 and total VASP protein expression in HCMECs. Cells were received HR injury (hypoxia 12/reoxygenation 12 hours) except control group and incubated with SCU (0.1, 1 and 10 μM) for 2 h prior to HR injury and following 24 h HR injury. The protein ratio was calculated by determining the band-integrated intensity that was normalized by that of GAPDH as a ratio of control. Data are means±SEM, n = 3 independent experiments with independent culture. One-way ANOVA followed by Fisher LSD test was used. *P<0.05, **P<0.01 and ***P<0.001 compared to HR group.

The effects of SCU on protein expression of PKG-I and phosphorylation of VASP under HR injury were shown in Fig 6. As shown in Fig 6A, SCU treatment still increased the protein levels of PKG-Iα under HR injury. And HR injury significantly decreased phosphorylation of VASP at Ser239 which reflected PKG enzyme activity, while SCU could keep the phosphorylation level of VASP against HR injury (Fig 6B).

**Fig 7:**
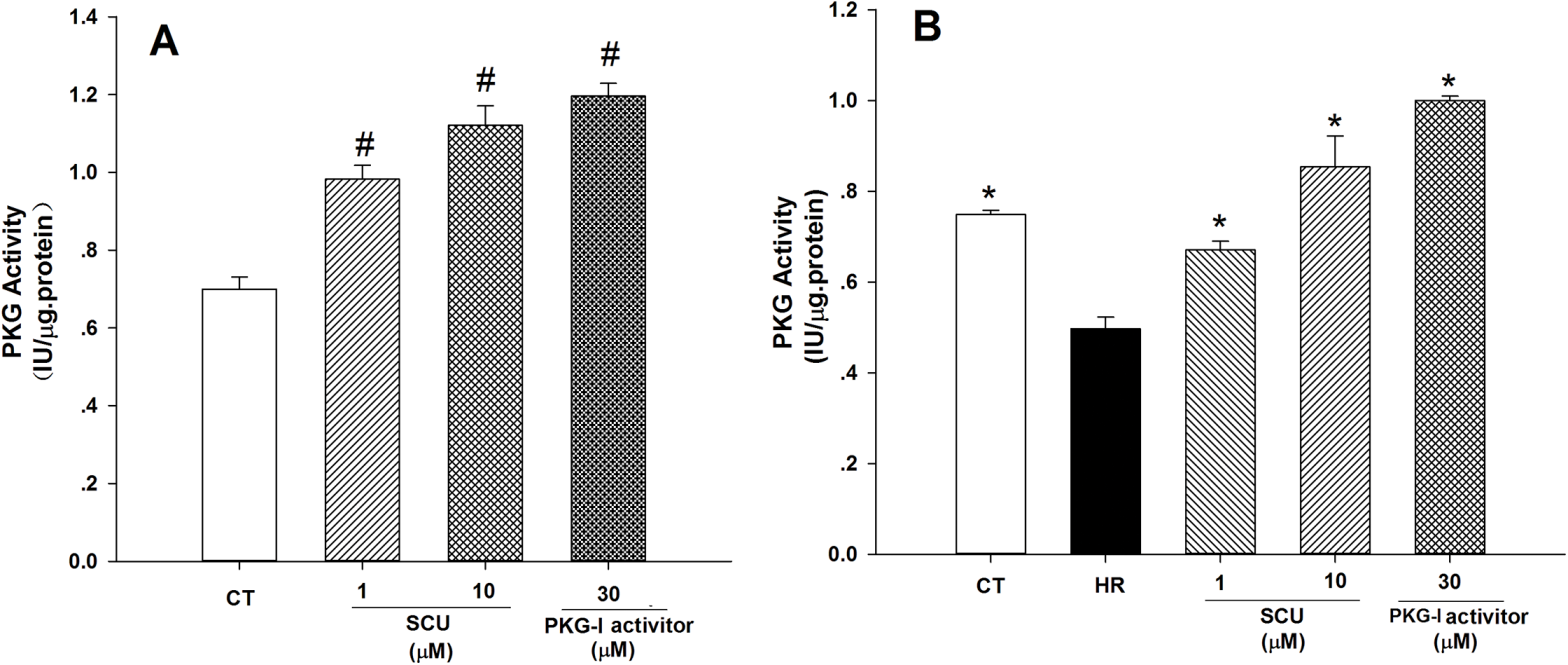
SCU increases PKG activity in normal and HR-treated HCMECs. (**A**) Experiment under normal culture condition: cells were incubated with SCU (1 and 10 μM) or PKG activator (8-pCPT-cGMP, 30μM, positive control) for 26 h except control group. (**B)** Experiment under HR condition: cells were received HR injury (hypoxia 12/reoxygenation 12 hours) except control group and incubated with SCU (1 and 10 μM) or PKG activator (8-pCPT-cGMP, 30μM) for 2 h prior to HR injury and following 24 h HR injury. PKG activity was measured by ELISA assay. Data are means±SEM, n = 3 independent experiments with independent culture. One-way ANOVA followed by the Fisher LSD test for comparisons was used, ^#^ *P*<0.05, *vs*. control (CT); *P<0.05, *vs*. HR group.

In addition, ELISA assay proved that SCU (1 and 10 μM) incubations significantly raised PKG activity in normal cultured HCMECs while PKG activator (8-pCPT-cGMP, 30 μM) used as positive control also increased PKG activity (Fig 7A). HR treatment significantly decreased PKG activity, and SCU incubations increased PKG activity against HR injury in HCMECs (Fig 7B).

### Phosphorylation states of PKG-Iα in HCMECs under different treatments

To clarify the role of PKG-Iα in IR injury and the protective mechanism of SCU, the phosphorylation states of PKG-Iα under normal and HR treatments were analyzed using MRM-MS (a targeted proteomic analysis method). PKG-Iα phosphopeptides with phosphorylation at Ser44, Ser50, Thr58, Ser64, Thr69, Ser72, Ser89 were detected. The PKG-Iα phosphopeptides were listed in S1 Table and the CID-MS/MS product ion spectrums of the phosphopeptides, which indicated the phosphorylation sites, were shown in S1 Fig Furthermore, the normalized peak areas of PKG-Iα phosphopeptides were shown in Table 2. As shown in Table 2, HR injury caused decrease in phosphorylation of PKG-Iα and the decrease was significant for phosphorylation at Ser50, Ser72, and Ser89. SCU (5 μM) treatment under HR condition could ameliorate the decrease in phosphorylation of PKG-Iα induced by HR injury. The phosphorylation of PKG-Iα at Ser44, Ser50, Thr58, Ser72 and Ser89 in HR+SCU group was significantly higher than that of HR group. The results suggested that HR injury inhibited the activation of PKG-Iα while SCU kept the activation state of PKG-Iα. In addition, two novel phosphorylation domains of PKG-Iα at Ser44 and Thr58 were found in our investigation and they had never been reported by any paper before.

**Table 2:** Normalized peak areas of PKG-I α phosphopeptides.

PKG I alpha phosphopeptides	Phosphorylation sites	Mean ± SEM				P value (T-test)		
		Control	SCU-treated	HR	HR+SCU	CT vs SCU	CT vs HR	HR vs HR+SCU
CQS[Pho]VLPVPSTHIGPRTTR	Phospho(S)@44	0.412±0.157	0.377±0.094	0.331±0.080	0.415±0.086	0.379	0.082	0.028*
CQSVLPVPS[Pho]THIGPRTTR	Phospho(S)@50	0.456±0.129	0.388±0.092	0.343±0.089	0.428±0.076	0.218	0.048*	0.046*
CQS[Pho]VLPVPS[Pho]THIGPRTTR	Phospho(S)@44@50	0.364±0.124	0.285±0.063	0.317±0.111	0.378±0.103	0.110	0.418	0.249
CQSVLPVPSTHIGPRTT[Pho]R	Phospho(T)@58	0.351±0.082	0.337±0.092	0.290±0.081	0.440±0.087	0.727	0.130	0.002*
AQGIS[Pho]AEPQTYR	Phospho(S)@64	0.353±0.099	0.315±0.113	0.322±0.100	0.347±0.123	0.451	0.514	0.643
AQGIS[Pho]AEPQT[Pho]YR	Phospho(S/T)@64@69	1.104±0.342	1.078±0.321	1.086±0.405	1.137±0.200	0.817	0.866	0.740
S[Pho]FHDLR	Phospho(S)@72	0.103±0.029	0.105±0.015	0.078±0.018	0.103±0.020	0.824	0.041*	0.010*
S[Pho]KDLIK	Phospho(S)@89	4.486±1.453	3.954±1.209	2.888±0.483	3.784±0.772	0.411	0.006*	0.009*

Cells of HCMECs were received HR injury (hypoxia 12/reoxygenation 12 hours) except control (CT) group and incubated with SCU (5 μM) for 2 h prior to HR injury and following 24 h HR injury. CT cells were cultured in parallel and kept in normal culture condition for the entire time period. n = 3 independent experiments with independent culture.

## Discussion

The present study demonstrated that SCU, a flavonoid glycoside successfully used in clinic in China for treatment of ischemic diseases, could protect endothelium function against MIR injury *in vivo*. In rats, MIR injury caused impairment in endothelium-dependent vasodilation, as assessed by ACH-induced dilation in isolated CA. A significant blunting of endothelium-mediated dilation was observed in MIR model group. The results were consistent to other reports supporting that IR injures endothelium and causes vascular ED [32–33]. SCU treatment not only decreased the myocardial infarct size caused by MIR, but also significantly augmented the endothelium-dependent relaxation in isolated coronary after MIR injury. The results that the protective effects of SCU could be ameliorated by PKG inhibitor Rp-8-Br-cGMP suggested the involvement of PKG pathway in the effects of SCU. Results of checking the phosphorylation of VASP at Ser239 suggested that SCU treatment induced increase in the level of p-VASP Ser239. Though VASP could be phosphorylated by both PKG and cAMP dependent protein kinase (PKA), phosphorylation at different sites such as phosphorylated-VASP at serine 239 (p-VASP Ser 239) and serine 157 (p-VASP Ser 157) were generated by PKG and PKA, respectively [34–35]. Therefore, level of p-VASP Ser239 could be used as an indicator of PKG activation. Activation of PKG might play an important role in SCU cardioprotective effects. Interestingly, a study using baicalin (an analog compound of SCU) also showed that the pharmacological effects of baicalin were related to PKG signal [36].

It is possible that the vasodilative effects of SCU, which had been shown in our previous study [21], might be involved in the protective effects of SCU against IR injury. While, in the present investigation, we focused on studying the possible direct cytoprotective effects of SCU on endothelial cells. In our experiments using cultured HCMECs, SCU protected cells against simulated HR injury. And, SCU induced both expression and activation of PKG-Iα in endothelial cells. ELISA assay of PKG activity proved that HR decreased PKG activity while SCU increased it.

PKG is a small family of eukaryotic serine/threonine kinases involved in the regulation of diverse physiological functions. Notably, PKG and its activator, cGMP, are well recognized modulators of cardiac function and stress response and it could serve as a myocardial brake to improve cell survival, protect against IR injury, etc [37–40]. Mammals possess two PKG genes, PKG-I and PKG-II, which encode for PKG-I protein (exits as a soluble homodimer) and PKG-II protein (exists as a monomer associated with membrane), respectively. And, alternate splicing of the same gene PKG-I generates two isoforms, PKG-Iα and PKG-Iβ [41]. The difference of PKG-I α and PKG-I β reside only in the N-terminal dimerization domain (about aa1-100) of the protein [42]. The role of PKG, especially PKG-Iα, in cell proliferation and cell survival was gradually recognized. Beside of acting as a key mediator of vasodilation, PKG-Iα plays an important role in preventing spontaneous apoptosis and promoting cell proliferation in both normal cells and certain cancer cells [43–48]. Basal or moderately elevated PKG-Iα activity could exhibit cytoprotective effects in many types of mammalian cells, including neural cells [46], human ovarian cancer cells [45, 49], primary murine vascular smooth muscle cells [50], and murine bone marrow stromal cells [44]. Small interfering RNA gene knockdown of PKG-Iα increased apoptosis and decreased proliferation in bone marrow stromal cells [44]. Therefore, the increased expression and activation of PKG-Iα induced by SCU treatment might contribute to the protective effects of SCU against IR injury. As a well-known direct scavenger of hydroxyl radicals, super oxide anion radicals and hydrogen peroxide [51–53], SCU might induce the transcription of PKG-Iα by decreasing the cellular level of reactive oxygen species. While, further studies clarifying the mechanism of the inducing effect of SCU on expression of PKG-Iα are necessary.

Since activation of PKG-Iα was based on its phosphorylation at specific sites, we studied the phosphorylation states of PKG-Iα of HCMECs under normal condition, HR injury, SCU treatment, HR + SCU treatment using a targeted proteomic technique. Beginning at the N terminus, PKG-I consists of the following functional domains in order of sequence: (aa1-116), the N-terminal dimerization/autoinhibitory domain; (aa117-234), the high-affinity cGMP binding domain; (aa235-355), the low-affinity cGMP binding domain; (aa356-491), the Mg^2+^/ATP binding site; (492–616), a target protein interaction domain; and (617–686), and C-terminal residues of unknown function [54]. The autoinhibitory domain plays a critical role in the activation of PKG because activation of PKG could be achieved by autophosphoryaltion in the autoinhibitory domain [54]. And, the difference between PKG-Iα and PKG-Iβ resides in the sequence at about aa1-100. Therefore, the phosphorylation state of the autophosphorylation domain (aa42-94) in PKG-Iα was thoroughly checked in the present study. The autophosphorylation sites of PKG-Iα had been studied before [54–55] but had not been fully clarified. In the present study, besides of reported phosphorylation sites of PKG-Iα such as Ser50, Ser72 and Ser89 [55], phosphorylation of PKG-Iα at Ser44 and Thr58 which had not been reported before were also detected. The detection of the new phosphorylation sites of PKG-Iα would be helpful to increase our knowledge about PKG-Iα and contribute to clarifying the function of PKG-Iα. Our targeted proteomic analysis also showed that, phosphorylation of PKG-Iα was inhibited by HR injury while SCU treatment could restore the phosphorylation of PKG-Iα. Significant decreases in phosphorylation of PKG-Iα at Ser50, Ser72 and Ser89 were found in HR injury group compared with control. SCU treatment under normal condition exhibited no significant influence on phosphorylation of PKG-Iα while SCU treatment under HR condition significantly increased the phosphorylation of PKG-Iα at Ser44, Thr58, Ser50, Ser72 and Ser89. Especially, the phosphorylation of PKG-Iα at Ser44 and Thr58, phosphorylation sites which had not been reported before, deserved further study. After activation, PKG-Iα could phosphorylate its downstream target proteins. Reported downstream targets of PKG-Iα in promoting cell survival and proliferation included VASP [44–45, 50], BAD [46], c-Src [45, 48], etc. VASP is a cytoskeletal protein which has diverse effects on cell motility, migration, and adhesion. Recent reports suggested the role of VASP in cell proliferation and cell survival. Phosphorylation of VASP could dampen MIR injury [56] as well as hepatic IR injury [57]. In the present study, the level of phosphorylated VASP (p-VASP) at Ser239 was also found to be increased in cells treated with SCU. The SCU-induced phosphorylation of VASP might play roles in the protective effects of SCU against MIR injury.

Actually, as animals’ individual differences and experimenters’ operation differences existing, there are some unavoidable reproducibility problems in the animal studies. In our MIR model surgery, different person usually produces model animal with different ischemia area, but for a well-trained experimenter, the differences caused can be kept minor. In order to avoid this difference as possible as we can, we just maintained the well-skilled operator to do the surgery, and tried to keep the same person to do the same experiment, so that we can keep smaller SEM in the same operation.

In summary, the present study demonstrates the protective effects of SCU on ED against MIR injury. The involvement of PKG pathway, especially the expression and phosphoryaltion of PKG-Iα, in the effects of SCU was observed. Result of the present study will shed new light on the mechanism study of SCU, a compound successfully used in clinic for ischemic diseases, also suggested the important role of PKG-Iα in IR-related signal cascades.

## Supporting Information

S1 Fig CID spectrum of phosphopeptides of PKG-I α. Highlighted in red circle are the site ions that lost 98 Da. (*A*) CQS[Pho]VLPVPSTHIGPRTTR, fragment ions b3-b18 suggested that the phosphorylation site might be on the S44,S50,T51,T57 or T58 while a loss of −98 Da at b3 confirmed the phosphorylation site to be S44. (*B*) CQSVLPVPS[Pho]THIGPRTTR, the CID-MS/MS product ion spectrum consisted of both b- and y-type ions, of which the fragment ions suggested that the phosphorylation site might be on the S44,S50,T51,T57 or T58 while a loss of −98 Da at b9 and y10 confirmed the phosphorylation site to be S50. (*C*) CQS[Pho]VLPVPS[Pho]THIGPRTTR, the CID-MS/MS product ion spectrum consisted of both b- and y-type ions, of which the fragment ions suggested that the phosphorylation site might be on the S44 and S50, while a loss of −98 Da at b3 and y10 confirmed the phosphorylation site to be S44 and S50. (*D*) CQSVLPVPSTHIGPRTT[Pho]R, the CID-MS/MS product ion spectrum consisted of both b- and y-type ions, of which the fragment ions suggested that the phosphorylation site might be on the S44,S50,T51,T57 or T58 while a loss of −98 Da at y2 confirmed the phosphorylation site to be T58. (*E*) AQGIS[Pho]AEPQTYR, fragment ions b5-b12 suggested that the phosphorylation site might be on the S64,T69 or Y70, while a loss of −98 Da at b5 confirmed the phosphorylation site to be S64. (*F*) AQGIS[Pho]AEPQT[Pho]YR, the CID-MS/MS product ion spectrum consisted of both b- and y-type ions, of which the fragment ions suggested that the phosphorylation site might be on the S64,T69 or Y70, while a loss of −98 Da at y3 and b5 confirmed the phosphorylation site to be S64,T69. (*G*) S[Pho]FHDLR, fragment ions b1–b4 or y1–y4 suggested that the phosphorylation site might be on the S72, while a neutral loss of −98 Da at b2 confirmed the phosphorylation site to be S89,the b- and y-type ions which were detected in the CID-MS/MS product ion spectrum were highlighted in red. (*H*) S[Pho]KDLIK, fragment ions b1–b5 or y6 suggested that the phosphorylation site might be on the S89, while a neutral loss of −98 Da at b5 confirmed the phosphorylation site to be S89, the b- and y-type ions which were detected in the CID-MS/MS product ion spectrum were highlighted in red.
(TIF)

S1 Table The phosphopeptides of PKG-I α detected in the MS analysis (DOC)

## References

[1] Zhang WD, Chen WS, Wang YH, Liu WY, Kong DY, Li HT. [Studies on flavone constituents of Erigeron breviscapus (Vant.) Hand.-Mazz]. China journal of Chinese materia medica. 2000;25: 536–538. 12516462

[2] Zhang Y, Wang X, Wang X, Xu Z, Liu Z, Ni Q, et al Protective effect of flavonoids from Scutellaria baicalensis Georgi on cerebral ischemia injury. J Ethnopharmacol. 2006;108: 355–360. 10.1016/j.jep.2006.05.022 16829002

[3] Hong H, Liu GQ. Scutellarin protects PC12 cells from oxidative stress-induced apoptosis. J Asian Nat Prod Res. 2007;9: 135–143. 10.1080/10286020412331286470 17479519

[4] Wang S, Wang H, Guo H, Kang L, Gao X, Hu L. Neuroprotection of Scutellarin is mediated by inhibition of microglial inflammatory activation. Neuroscience. 2011;185:150–160. 10.1016/j.neuroscience.2011.04.005 21524691

[5] Pan Z, Feng T, Shan L, Cai B, Chu W, Niu H, et al Scutellarin-induced endothelium-independent relaxation in rat aorta. Phytother Res: PTR. 2008;22: 1428–1433. 10.1002/ptr.2364 18972583

[6] Dai H, Gu J, Li LZ, Yang LM, Liu H, Li JY. Scutellarin benzyl ester partially secured the ischemic injury by its anti-apoptosis mechanism in cardiomyocytes of neonatal rats. Journal of Chinese integrative medicine. 2011;9: 1014–1021. 10.3736/jcim20110913 21906527

[7] Zhang HF, Hu XM, Wang LX, Xu SQ, Zeng FD. Protective effects of scutellarin against cerebral ischemia in rats: evidence for inhibition of the apoptosis-inducing factor pathway. Planta Med. 2009;75:121–126. 10.1055/s-0028-1088368 19031363

[8] Gao ZX, Huang DY, Li HX, Zhang LN, Lv YH, Cui HD, et al Scutellarin promotes in vitro angiogenesis in human umbilical vein endothelial cells. Biochem Biophys Res Commun. 2010;400: 151–156. 10.1016/j.bbrc.2010.08.034 20709020

[9] Lin LL, Liu AJ, Liu JG, Yu XH, Qin LP, Su DF. Protective effects of scutellarin and breviscapine on brain and heart ischemia in rats. J Cardiovasc Pharmacol. 2007;50: 327–332. 10.1097/FJC.0b013e3180cbd0e7 17878763

[10] Radovits T, Zotkina J, Lin LN, Karck M, Szabo G. Endothelial dysfunction after hypoxia-reoxygenation: do in vitro models work? Vascul Pharmacol. 2009;51: 37–43. 10.1016/j.vph.2009.01.009 19275967

[11] Eltzschig HK, Collard CD. Vascular ischaemia and reperfusion injury. Br Med Bull. 2004;70: 71–86. 10.1093/bmb/ldh025 15494470

[12] Laude K, Beauchamp P, Thuillez C, Richard V. Endothelial protective effects of preconditioning. Cardiovasc Res. 2002;55: 466–473. 10.1016/S0008-6363(02)00277-8 12160943

[13] Carden DL, Granger DN. Pathophysiology of ischaemia-reperfusion injury. J Pathol. 2000;190: 255–266. 10.1002/(SICI)1096-9896(200002)190:3<255::AID-PATH526>3.0.CO;2-6 10685060

[14] Vinten-Johansen J, Zhao ZQ, Nakamura M, Jordan JE, Ronson RS, Thourani VH, et al Nitric oxide and the vascular endothelium in myocardial ischemia-reperfusion injury. Ann N Y Acad Sci. 1999;874: 354–370. 10.1111/j.1749-6632.1999.tb09251.x 10415547

[15] Turer AT, Hill JA. Pathogenesis of myocardial ischemia-reperfusion injury and rationale for therapy. Am J Cardiol. 2010;106: 360–368. 10.1016/j.amjcard.2010.03.032 20643246 PMC2957093

[16] Harrison DG. Endothelial function and oxidant stress. Clin Cardiol. 1997;20: II-11-17. 9422847 10.1002/j.1932-8737.1997.tb00007.x PMC7509624

[17] Kharbanda RK, Peters M, Walton B, Kattenhorn M, Mullen M, Klein N, et al Ischemic preconditioning prevents endothelial injury and systemic neutrophil activation during ischemia-reperfusion in humans in vivo. Circulation. 2001;103: 1624–1630. 10.1161/01.CIR.103.12.1624 11273988

[18] Bucci M, Papapetropoulos A, Vellecco V, Zhou Z, Zaid A, Giannogonas P, et al cGMP-dependent protein kinase contributes to hydrogen sulfide-stimulated vasorelaxation. PloS one. 2012;7: e53319 10.1371/journal.pone.0053319 23285278 PMC3532056

[19] Qin X, Zheng X, Qi H, Dou D, Raj JU, Gao Y. cGMP-dependent protein kinase in regulation of basal tone and in nitroglycerin- and nitric-oxide-induced relaxation in porcine coronary artery. Pflugers Arch. 2007;454: 913–923. 10.1007/s00424-007-0249-8 17377806

[20] Yao X, Kwan HY, Chan FL, Chan NW, Huang Y. A protein kinase G-sensitive channel mediates flow-induced Ca(2+) entry into vascular endothelial cells. FASEB J. 2000;14: 932–938. 10783147 10.1096/fasebj.14.7.932

[21] Yang W, Lust RM, Bofferding A, Wingard CJ. Nitric oxide and catalase-sensitive relaxation by scutellarin in the mouse thoracic aorta. J Cardiovasc Pharmacol. 2009;53: 66–76. 10.1097/FJC.0b013e318195d776 19129733

[22] Hou Y, Lascola J, Dulin NO, Ye RD, Browning DD. Activation of cGMP-dependent protein kinase by protein kinase C. J Biol Chem. 2003;278: 16706–16712. 10.1074/jbc.M300045200 12609995

[23] Chu DM, Francis SH, Thomas JW, Maksymovitch EA, Fosler M, Corbin JD. Activation by autophosphorylation or cGMP binding produces a similar apparent conformational change in cGMP-dependent protein kinase. J Biol Chem. 1998;273: 14649–14656. 10.1074/jbc.273.23.14649 9603983

[24] Landgraf W, Hullin R, Gobel C, Hofmann F. Phosphorylation of cGMP-dependent protein kinase increases the affinity for cyclic AMP. Eur J Biochem. 1986;154: 113–117. 10.1111/j.1432-1033.1986.tb09365.x 3002787

[25] Hofmann F, Gensheimer HP, Gobel C. cGMP-dependent protein kinase. Autophosphorylation changes the characteristics of binding site 1. Eur J Biochem. 1985;147: 361–365. 10.1111/j.1432-1033.1985.tb08758.x 2982615

[26] Riederer A, Held B, Mack B. [Immunohistochemical study of the distribution of constitutive nitric oxide synthase in vascular endothelium of the nasal mucosa in the human]. Laryngorhinootologie. 1999;78: 373–377. 10.1055/s-2007-996889 10457518

[27] Inserte J, Garcia-Dorado D. cGMP/PKG Pathway as a common mediator of cardioprotection. Translatability and mechanism. Br J Pharmacol. 2015 4;172(8):1996–2009. 10.1111/bph.12959 25297462 PMC4386977

[28] Inserte J, Hernando V, Vilardosa U, Abad E, Poncelas-Nozal M, Garcia-Dorado D. Activation of cGMP/protein kinase G pathway in postconditioned myocardium depends on reduced oxidative stress and preserved endothelial nitric oxide synthase coupling. J Am Heart Assoc. 2013;2: e005975 10.1161/JAHA.112.005975 23525447 PMC3603241

[29] Burley DS, Ferdinandy P, Baxter GF. Cyclic GMP and protein kinase-G in myocardial ischaemia-reperfusion: opportunities and obstacles for survival signaling. Br J Pharmacol. 2007;152: 855–869. 10.1038/sj.bjp.0707409 17700722 PMC2078226

[30] Das A, Smolenski A, Lohmann SM, Kukreja RC. Cyclic GMP-dependent protein kinase Ialpha attenuates necrosis and apoptosis following ischemia/reoxygenation in adult cardiomyocyte. J Biol Chem. 2006;281: 38644–38652. 10.1074/jbc.M606142200 17038326

[31] Costa AD, Pierre SV, Cohen MV, Downey JM, Garlid KD. cGMP signalling in pre- and post-conditioning: the role of mitochondria. Cardiovasc Res. 2008;77: 344–352. 10.1093/cvr/cvm050 18006449

[32] Deanfield JE, Halcox JP, Rabelink TJ. Endothelial function and dysfunction: testing and clinical relevance. Circulation. 2007;115: 1285–1295. 10.1161/CIRCULATIONAHA.106.652859 17353456

[33] Friedewald VE, Giles TD, Pool JL, Yancy CW, Roberts WC. The Editor's Roundtable: Endothelial dysfunction in cardiovascular disease. Am J Cardiol. 2008;102: 418–423. 10.1016/j.amjcard.2008.05.005 18678298

[34] Chen H, Levine YC, Golan DE, Michel T, Lin AJ. Atrial natriuretic peptide-initiated cGMP pathways regulate vasodilator-stimulated phosphoprotein phosphorylation and angiogenesis in vascular endothelium. J Biol Chem. 2008;283: 4439–4447. 10.1074/jbc.M709439200 18079117

[35] Schäfer A, Burkhardt M, Vollkommer T, Bauersachs J, Munzel T, Walter U, et al Endothelium-dependent and -independent relaxation and VASP serines 157/239 phosphorylation by cyclic nucleotide-elevating vasodilators in rat aorta. Biochem Pharmacol. 2003;65: 397–405. 10.1016/S0006-2952(02)01523-X 12527332

[36] Lin YL, Dai ZK, Lin RJ, Chu KS, Chen IJ, Wu JR, et al Baicalin, a flavonoid from Scutellaria baicalensis Georgi, activates large-conductance Ca2+-activated K+ channels via cyclic nucleotide-dependent protein kinases in mesenteric artery. Phytomedicine. 2010;17: 760–770. 10.1016/j.phymed.2010.01.003 20171070

[37] Zhang M, Kass DA. Phosphodiesterases and cardiac cGMP: evolving roles and controversies. Trends Pharmacol Sci. 2011;32: 360–365. 10.1016/j.tips.2011.02.019 21477871 PMC3106121

[38] Heiss C, Keen CL, Kelm M. Flavanols and cardiovascular disease prevention. Eur Heart J. 2010;31: 2583–2592. 10.1093/eurheartj/ehq332 20852295

[39] Frelinger AL 3rd, Jakubowski JA, Brooks JK, Carmichael SL, Berny-Lang MA, Barnard MR, et al Platelet activation and inhibition in sickle cell disease (pains) study. Platelets. 2014; 25: 27–35. 10.3109/09537104.2013.770136 23469943

[40] Chang AC, Patenaude A, Lu K, Fuller M, Ly M, Kyle A, et al Notch-dependent regulation of the ischemic vasodilatory response–-brief report. Arterioscler Thromb Vasc Biol. 2013;33: 510–512. 10.1161/ATVBAHA.112.300840 23288167

[41] Abdallah Y, Gkatzoflia A, Pieper H, Zoga E, Walther S, Kasseckert S, et al Mechanism of cGMP-mediated protection in a cellular model of myocardial reperfusion injury. Cardiovasc Res. 2005;66: 123–131. 10.1016/j.cardiores.2005.01.007 15769455

[42] Francis SH, Woodford TA, Wolfe L, Corbin JD. Types I alpha and I beta isozymes of cGMP-dependent protein kinase: alternative mRNA splicing may produce different inhibitory domains. Second messengers and phosphoproteins. 1988;12: 301–310. 3272299

[43] Marathe N, Rangaswami H, Zhuang S, Boss GR, Pilz RB. Pro-survival effects of 17beta-estradiol on osteocytes are mediated by nitric oxide/cGMP via differential actions of cGMP-dependent protein kinases I and II. J Biol Chem. 2012;287: 978–988. 10.1074/jbc.M111.294959 22117068 PMC3256896

[44] Wong JC, Fiscus RR. Essential roles of the nitric oxide (no)/cGMP/protein kinase G type-Ialpha (PKG-Ialpha) signaling pathway and the atrial natriuretic peptide (ANP)/cGMP/PKG-Ialpha autocrine loop in promoting proliferation and cell survival of OP9 bone marrow stromal cells. J Cell Biochem. 2011;112: 829–839. 10.1002/jcb.22981 21328456

[45] Leung EL, Wong JC, Johlfs MG, Tsang BK, Fiscus RR. Protein kinase G type Ialpha activity in human ovarian cancer cells significantly contributes to enhanced Src activation and DNA synthesis/cell proliferation. Mol Cancer Res. 2010;8: 578–591. 10.1158/1541-7786.MCR-09-0178 20371672

[46] Johlfs MG, Fiscus RR. Protein kinase G type-Ialpha phosphorylates the apoptosis-regulating protein Bad at serine 155 and protects against apoptosis in N1E-115 cells. Neurochem Int. 2010;56: 546–553. 10.1016/j.neuint.2009.12.017 20043968

[47] Fiscus RR. Involvement of cyclic GMP and protein kinase G in the regulation of apoptosis and survival in neural cells. Neurosignals. 2002;11: 175–190. 10.1159/000065431 12393944

[48] Wong JC, Bathina M, Fiscus RR. Cyclic GMP/protein kinase G type-Ialpha (PKG-Ialpha) signaling pathway promotes CREB phosphorylation and maintains higher c-IAP1, livin, survivin, and Mcl–1 expression and the inhibition of PKG-Ialpha kinase activity synergizes with cisplatin in non-small cell lung cancer cells. J Cell Biochem. 2012;113: 3587–3598. 10.1002/jcb.24237 22740515

[49] Fraser M, Chan SL, Chan SS, Fiscus RR, Tsang BK. Regulation of p53 and suppression of apoptosis by the soluble guanylyl cyclase/cGMP pathway in human ovarian cancer cells. Oncogene. 2006;25: 2203–2212. 10.1038/sj.onc.1209251 16288207

[50] Wong JC, Fiscus RR. Protein kinase G activity prevents pathological-level nitric oxide-induced apoptosis and promotes DNA synthesis/cell proliferation in vascular smooth muscle cells. Cardiovasc Pathol. 2010;19: e221–231. 10.1016/j.carpath.2009.11.001 20060325

[51] Qian LH, Li NG, Tang YP, Zhang L, Tang H, Wang ZJ, et al Synthesis and bio-activity evaluation of scutellarein as a potent agent for the therapy of ischemic cerebrovascular disease. Int J Mol Sci. 2011;12: 8208–8216. 10.3390/ijms12118208 22174659 PMC3233465

[52] Liu H, Yang XL, Wang Y, Tang XQ, Jiang DY, Xu HB. Protective effects of scutellarin on superoxide-induced oxidative stress in rat cortical synaptosomes. Acta Pharmacol Sin. 2003;24: 1113–1117. 14627495

[53] Liu H, Yang X, Zhou L, Xu H. [Study on effects of scutellarin on scavenging reactive oxygen]. Journal of Chinese medicinal materials. 2002;25: 491–493. 12599762

[54] Wall ME, Francis SH, Corbin JD, Grimes K, Richie-Jannetta R, Kotera J, et al Mechanisms associated with cGMP binding and activation of cGMP-dependent protein kinase. Proc Natl Acad Sci U S A. 2003;100: 2380–2385. 10.1073/pnas.0534892100 12591946 PMC151349

[55] Busch JL, Bessay EP, Francis SH, Corbin JD. A conserved serine juxtaposed to the pseudosubstrate site of type I cGMP-dependent protein kinase contributes strongly to autoinhibition and lower cGMP affinity. J Biol Chem. 2002;277: 34048–34054. 10.1074/jbc.M202761200 12080049

[56] Köhler D, Straub A, Weissmüller T, Faigle M, Bender S, Lehmann R, et al Phosphorylation of vasodilator-stimulated phosphoprotein prevents platelet-neutrophil complex formation and dampens myocardial ischemia-reperfusion injury. Circulation. 2011;123: 2579–2590. 10.1161/CIRCULATIONAHA.110.014555 21606399

[57] Köhler D, Birk P, König K, Straub A Eldh T, Morote-Garcia JC, et al Phosphorylation of vasodilator-stimulated phosphoprotein (VASP) dampens hepatic ischemia-reperfusion injury. PloS one. 2011;6: e29494 10.1371/journal.pone.0029494 22216296 PMC3245274

