# Supplementary figures and images for: Scutellarin’s Cardiovascular Endothelium Protective Mechanism: Important Role of PKG-Iα
Source: PLoS One. 2015 Oct 6;10(10):e0139570. doi: 10.1371/journal.pone.0139570 (PMC4594915; doi:10.1371/journal.pone.0139570)

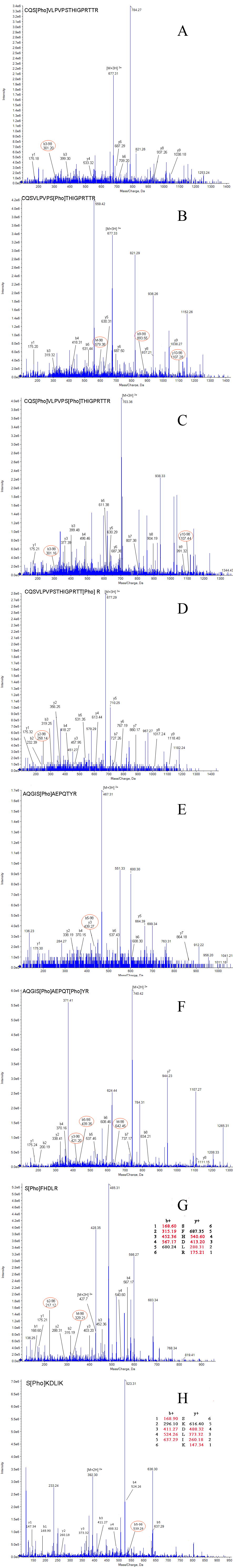

Supplement: S1 Fig — Highlighted in red circle are the site ions that lost 98 Da. (A) CQS[Pho]VLPVPSTHIGPRTTR, fragment ions b3-b18 suggested that the phosphorylation site might be on the S44,S50,T51,T57 or T58 while a loss of −98 Da at b3 confirmed the phosphorylation site to be S44. (B) CQSVLPVPS[Pho]THIGPRTTR, the CID-MS/MS product ion spectrum consisted of both b- and y-type ions, of which the fragment ions suggested that the phosphorylation site might be on the S44,S50,T51,T57 or T58 while a loss of −98 Da at b9 and y10 confirmed the phosphorylation site to be S50. (C) CQS[Pho]VLPVPS[Pho]THIGPRTTR, the CID-MS/MS product ion spectrum consisted of both b- and y-type ions, of which the fragment ions suggested that the phosphorylation site might be on the S44 and S50, while a loss of −98 Da at b3 and y10 confirmed the phosphorylation site to be S44 and S50. (D) CQSVLPVPSTHIGPRTT[Pho]R, the CID-MS/MS product ion spectrum consisted of both b- and y-type ions, of which the fragment ions suggested that the phosphorylation site might be on the S44,S50,T51,T57 or T58 while a loss of −98 Da at y2 confirmed the phosphorylation site to be T58. (E) AQGIS[Pho]AEPQTYR, fragment ions b5-b12 suggested that the phosphorylation site might be on the S64,T69 or Y70, while a loss of −98 Da at b5 confirmed the phosphorylation site to be S64. (F) AQGIS[Pho]AEPQT[Pho]YR, the CID-MS/MS product ion spectrum consisted of both b- and y-type ions, of which the fragment ions suggested that the phosphorylation site might be on the S64,T69 or Y70, while a loss of −98 Da at y3 and b5 confirmed the phosphorylation site to be S64,T69. (G) S[Pho]FHDLR, fragment ions b1–b4 or y1–y4 suggested that the phosphorylation site might be on the S72, while a neutral loss of −98 Da at b2 confirmed the phosphorylation site to be S89,the b- and y-type ions which were detected in the CID-MS/MS product ion spectrum were highlighted in red. (H) S[Pho]KDLIK, fragment ions b1–b5 or y6 suggested that the phosphorylation site might be on [file pone.0139570.s001.tif]
