# Supplementary material for: Scutellarin’s Cardiovascular Endothelium Protective Mechanism: Important Role of PKG-Iα
Source: PLoS One. 2015 Oct 6;10(10):e0139570. doi: 10.1371/journal.pone.0139570 (PMC4594915; doi:10.1371/journal.pone.0139570)
Supplement: S1 Table — (DOC) [file pone.0139570.s002.doc]

**S1 Table:**

**S1Table. The phosphopeptides of PKG-I α detected in the MS** analysis

| Sequence | Modifications | Prec MW | z |
| --- | --- | --- | --- |
| CQS[Pho]VLPVPSTHIGPRTTR | Phospho(S)@44 | 2028 | 3 |
| CQSVLPVPS[Pho]THIGPRTTR | Phospho(S)@50 | 2028 | 3 |
| CQS[Pho]VLPVPS[Pho]THIGPRTTR | Phospho(S)@44@50 | 2108 | 3 |
| CQSVLPVPSTHIGPRTT[Pho]R | Phospho(T)@58 | 2028 | 3 |
| AQGIS[Pho]AEPQTYR | Phospho(S)@64 | 1399.6 | 3 |
| AQGIS[Pho]AEPQT[Pho]YR | Phospho(S/T)@64@69 | 1479.6 | 2 |
| S[Pho]FHDLR | Phospho(S)@72 | 853.3 | 2 |
| S[Pho]KDLIK | Phospho(S)@89 | 782.4 | 2 |
